# ProtoBug: functional families from the complete proteomes of insects

**DOI:** 10.1093/database/bau122

**Published:** 2015-04-24

**Authors:** Nadav Rappoport, Michal Linial

**Affiliations:** ^1^School of Computer Science and Engineering and ^2^Department of Biological Chemistry, Institute of Life Sciences, The Hebrew University of Jerusalem, Givat Ram Campus, Jerusalem, 91904 Israel

## Abstract

ProtoBug (http://www.protobug.cs.huji.ac.il) is a database and resource of protein families in Arthropod genomes. ProtoBug platform presents the relatedness of complete proteomes from 17 insects as well as a proteome of the crustacean, *Daphnia pulex*. The represented proteomes from insects include louse, bee, beetle, ants, flies and mosquitoes. Based on an unsupervised clustering method, protein sequences were clustered into a hierarchical tree, called ProtoBug. ProtoBug covers about 300 000 sequences that are partitioned to families. At the default setting, all sequences are partitioned to ∼20 000 families (excluding singletons). From the species perspective, each of the 18 analysed proteomes is composed of 5000–8000 families. In the regime of the advanced operational mode, the ProtoBug provides rich navigation capabilities for touring the hierarchy of the families at any selected resolution. A proteome viewer shows the composition of sequences from any of the 18 analysed proteomes. Using functional annotation from an expert system (Pfam) we assigned domains, families and repeats by 4400 keywords that cover 73% of the sequences. A strict inference protocol is applied for expanding the functional knowledge. Consequently, secured annotations were associated with 81% of the proteins, and with 70% of the families (≥10 proteins each). ProtoBug is a database and webtool with rich visualization and navigation tools. The properties of each family in relation to other families in the ProtoBug tree, and in view of the taxonomy composition are reported. Furthermore, the user can paste its own sequences to find relatedness to any of the ProtoBug families. The database and the navigation tools are the basis for functional discoveries that span 350 million years of evolution of Arthropods. ProtoBug is available with no restriction at: www.protobug.cs.huji.ac.il.

**Database URL:** www.protobug.cs.huji.ac.il.

## Introduction

With the maturation of sequencing technologies, we now have a large number of completely sequenced genomes. Recent sequencing efforts have yielded a large and diverse collection of complete genomes of insects. The genome and proteome of *Apis mellifera* provide a glimpse on the first social insect (1). Several ant representatives have been recently sequenced (2). Ants comprise the largest and most diverse group within Hymenoptera (3). In analysing a newly sequenced genome, the immediate task is to determine the protein sequences and their genomic borders (4). A more challenging task is to reliably assign functional annotations to predicted coding sequences. Major resources for functional annotations include UniProtKB keywords (5), Pfam (families, domains and repeats) (6), Gene Ontology (7) and the unified resource of InterPro (8). The success in annotated proteins at a proteome scale using unsupervised clustering was illustrated for *A. mellifera* (9) and *Daphnia pulex* (10). The quality of automatic methods for assigning functions to uncharacterized proteins is extensively discussed (11). ProtoBug 1.0 resource clusters protein sequences from Arthropods to functional families. The approach is based on the ProtoNet algorithm (12) that was designed to cope with a large number of sequences using an efficient and accurate algorithm (13). ProtoNet algorithm is automatic, unsupervised clustering algorithm, which groups proteins according to their mutual sequence similarity. The clustering does not rely on multiple sequence alignments and the input consists raw sequences with no additional knowledge.

The ProtoBug database classifies all 17 completely sequenced insect proteomes as well as one crustacean (*D. pulex*). It provides a rich resource for comparative perspective of protein families of all insects’ proteomes. An interactive searching of protein families and tracing their evolutionary relatedness are available at http://www.proto bug.cs.huji.ac.il.

## Constructing ProtoBug Tree

### Data resources

The protein sequences that are included in ProtoBug platform include only completely sequenced genomes. Several criteria were applied to compile the protein sequence collection: (i) A minimal quality for proteomes’ completeness. We set a minimum of 10 000 sequences as a threshold; (ii) A wide range of divergence time among Neoptera. The human body louse is a prototype for aphids, shield bugs and cicadas (belong to Hemiptera). We also included the *D. pulex* as outgroup for insects. (iii) Reduced redundancy. We selected only 2 complete proteomes out of the 12 species of the Drosophilae. The model organisms *Drosophila melanogaster* covers related species (*Drosophila simulans* and *Drosophila sechellia*). We also selected the *Drosophila* *virilis* that is separated by about 40 M years of evolution from the *D. melanogaster*. (iv) Including fast evolving insects. We included the ants in the list of complete proteomes to cover a range of habitats, ecological needs, sizes and social behaviors.

We downloaded the whole proteome of *Tribolium castaneum*, *Aedes aegypti, Anopheles gambiae, Culex quinquefasciatus, D.* *melanogaster, D. virilis, Solenopsis invicta, Anopheles darlingi, Acromyrmex echinatior, Camponotus floridanus, Pediculus humanus, Harpegnathos saltator* and *D. pulex* from UniProtKB (14). Other proteomes were downloaded directly from the Hymenoptera Genome Database (HGD) (15): *Nasonia vitripennis* (v1.2), *Linepithema humile* (v1.2), *Atta cephalotes* (v1.2), *Pogonomyrmex barbatus* (v1.2) and *A. mellifera* (release 4.5).

Table 1 summarizes the statistics of the analysed species. Note that *D. pulex* (16) was added as an outgroup species. The total number of proteins from the combined resources is 287 405 (206 615 from UniProtKB and 80 790 from HGD). There are 138 762 proteins that belong to the Hymenoptera (48%) and 91 241 (32%) to the Diptera.

**Table 1.** Collection of 18 Arthropods proteomes

Taxa-ID	Organism (common name)	DB Sourcea	No. Proteins	No. Fam	No. TMD’s proteinsc
7070	*T. castaneum* (Red flour beetle)	UniProt	16502	6706	3670
7159	*Ae. aegypti* (Yellowfever mosquito)	UniProt	16045	5120	3442
7165	*An. gambiae* (African malaria mosquito)	UniProt	13075	4563	3101
7176	*Cx. quinquefasciatus* (Southern house mosquito)	UniProt	18703	5415	3713
7227	*D. melanogaster* (Fruit fly)	UniProt	17524	5233	4277
7244	*D. virilis* (Fruit fly)	UniProt	14457	5662	3202
7425	*N. vitripennis* (Parasitic wasp)	HGD (v1.2)	18822	5058	3128
13686	*S. invicta* (Red imported fire ant)	UniProt	14194	5814	2669
43151	*An. darlingi* (Mosquito)	UniProt	11437	5446	2407
103372	*Acromyrmex echinatior* (Panamanian leafcutter ant)	UniProt	13962	6538	2812
104421	*C. floridanus* (Florida carpenter ant)	UniProt	14787	6008	2983
121224	*P. humanus subsp. corporis* (Body louse)	UniProt	10763	4866	2451
610380	*H. saltator* (Jumping Ant)	UniProt	15029	5423	2768
7460	*A. mellifera* (Honeybee)	BeeBase (v4.5)	10570	4521	2561
83485	*L. humile* (Argentine ant)	HGD (v1.2)	16116	6964	3290
12957	*A. cephalotes* (Leaf Cutter Ant)	HGD (v1.2)	18093	8378	3777
144034	*P. barbatus* (Red Harvester Ant)	HGD (v1.2)	17189	7999	3530
6669	*D. pulex* (Water flea)	UniProt	30137	8742	4770

^a^Source of BeeBase belong to HGD, Hymenoptera Genome Database.

^b^Short proteins are ≤100 aa.

^c^Number of proteins predicted as having TMDs.

### Hierarchical clustering algorithm

The clustering protocol starts by pre-calculating an all-against-all BLAST similarity score (17) for all 300 000 protein sequences. The similarities’ E-scores were used to produce a continuous hierarchical bottom-up clustering process. At each step, the two most similar protein clusters are joined [the exact algorithm is described in reference (13)]. Importantly, BLAST E-score with an extremely relaxed threshold is considered throughout the tree construction (E-score = 100). The agglomerative clustering algorithm benefits from such relaxed E-score distances for constructing a robust family tree. A key ingredient of ProtoBug is the use of a Constrained Memory-ProtoNet algorithm (13). The algorithm has previously described for the ProtoNet platform (12, 18).

In the clustering tree, there is a natural tradeoff of the number of clusters, clusters’ size and their quality. The result is a hierarchy of protein clusters at various degrees of granularity. This hierarchy is structured as a collection of trees that form what we call—the ProtoBug forest. The root clusters contain all the proteins of the tree while other clusters represent subdivisions of proteins into smaller groups. The hierarchical definitions allow the user to navigate from a protein to the sub-family and the super-family levels in order to discover overlooked functions and evolutionary signals.

### Protein families

**Figure 1.**
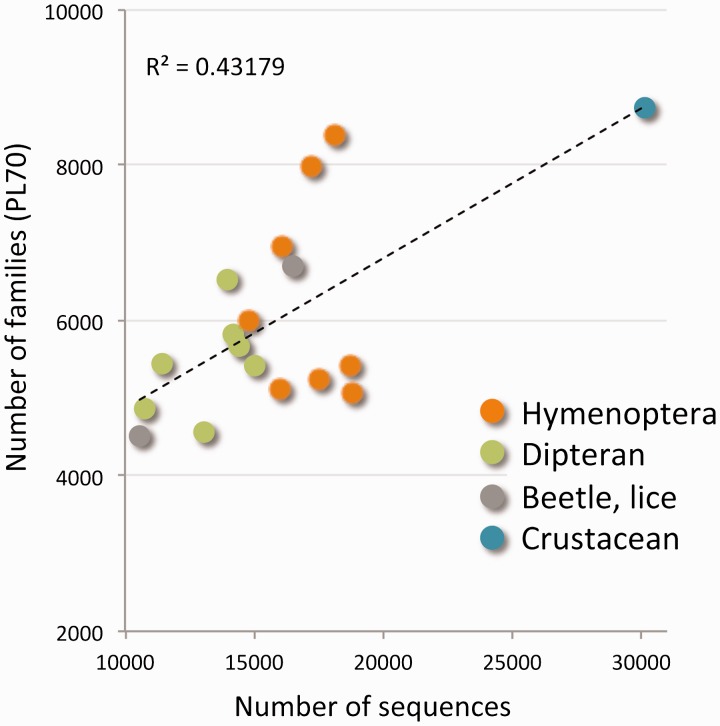
Protein families of the Arthropods complete proteomes. The scatter plot shows the number of protein families from the ProtoBug tree with respect to the number of raw sequences for each of the 18 analysed proteomes. The families are disjoint clusters from the partition at PL70. Although some organisms appear in >8000 families, most organisms participate in 5000–5500 protein families. The organisms are colored by the main clades. The extreme value of ∼30 000 proteins belongs to *D. pulex*.

The tree of functional families is naturally leveled according to the percentage of merging steps carried out so far (ProtoLevel). We sought the level that provides the best partition into functional families. This default level (or cut) turned out to be ProtoLevel70 (PL70). Using the PL70 threshold we obtained 20 134 disjoint clusters that represent protein families. Figure 1 shows the number of protein families and the correlation to the number of protein sequences for each of the 18 species. There are 6000 families per genome on average. Actually, the range for the number of families per genome is quite broad (between 4500 and 8000). The variability of protein numbers is monitored for the two major clades of insects: the Hymenoptera and the Diptera. The high number of families in *D. pulex* is in accord with the exceptional number of proteins (over 30 000 sequences).

As the clustering relies on complete proteomes, we take advantage of the expectation for appearance of proteins for a family from each of the tested organisms (Table 1). Analysis of the ProtoBug algorithm reveals that the average number of species for PL70 clusters is 15.5. Estimating the random expectation (fixing the number of proteins from each species, and according to the multinomial distribution) is only 12. The *P*-value of having 15 species (or more) in a random cluster of size 18 is significant (*P* = 0.00059). Thus, the PL70 level leads to a good representation of all the analysed insects. The rest of the analyses will refer to the 20 134 clusters from the PL70 threshold as disjoint protein families. Each family is associated with a ProtoBug identifier.

**Figure 2.**
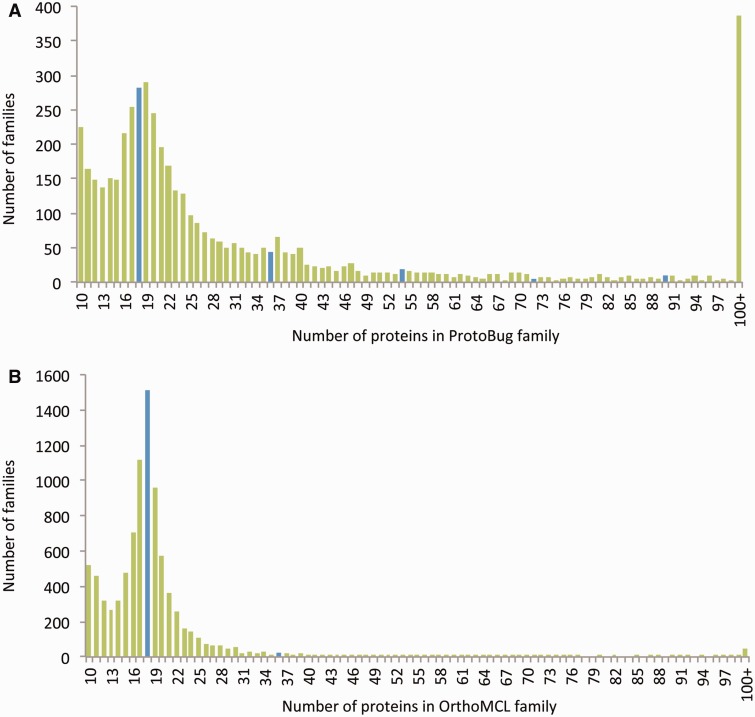
Size distribution of the protein families from Arthropods-complete proteomes. The families listed are based on all 18 complete proteomes. The protein families are ranked by their sizes according to ProtoBug clusters **(A)** and OrthoMCL **(B)** algorithms. The blue bars shows the families of size 18 and the multiplications (i.e. 36, 54, etc.). Note a clear difference in cluster size distribution between the two clustering modes. Specifically, there are ∼400 families with more than 100 proteins among the ProtoBug family collection.

For comparison, we applied the OrthoMCL clustering algorithm on the same set of sequences (19). The result from both algorithms is a comparable number of families (the number is 26 204 for OrthoMCL) (Figure 2). The number of proteins that were clustered to small clusters of size 3 or less by OrthoMCL and ProtoBug are ∼25 K and ∼27 K, respectively. However, the cluster size that was obtained by applying OrthoMCL is substantially smaller (Figure 2A and B). The largest difference between the two alternative classification modes is associated with the largest families. In ProtoNet based clusters, there are 383 clusters of size >100 proteins (a total of 118 534 proteins) relative to only 45 large clusters by OrthoMCL (total of 6933 proteins). Although 41% of all proteins are included in large ProtoBug clusters (>100 proteins), only 2.4% of the proteins meet that size for the OrthoMCL clusters (Figure 2).

### Quality of families

The protocol that constructs ProtoBug is unsupervised. Therefore, no annotations are included. However, measuring the correspondence between a given cluster and specific annotations provided by external expert systems is essential for the ‘validation’ of the quality of the automatically generated families. Each protein was assigned with its predicted Pfam keywords (domains, families and repeats), leaving 77 988 (27% of all the sequences) unannotated. Altogether there are 4400 Pfam keywords that were assigned to the analysed proteomes. Based on a minimal threshold for the family size, 3437 Pfam keywords are associated with 4504 families (≥10 proteins each).

**Figure 3.**
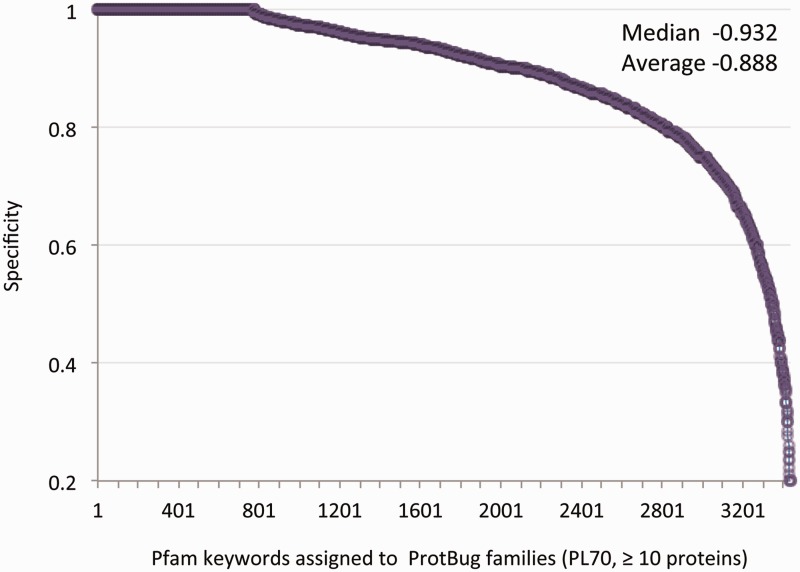
Specificity for all annotated families. Each data-point represents a unique annotation from a set of Pfam keywords. There are 3437 Pfam keywords that are associated with 4504 families (>10 proteins each). The annotation inference is restricted to a minimal specificity of 0.2. The average and median specificity are shown.

At the granularity level of PL70, many families are pure and sensitive in term of their associated keywords. For example, Trypsin (2912 proteins, no false positives) and Ras (1559 proteins, one false positive) are confined to only a single family. Nevertheless, many keywords appear in more than one family (i.e. low sensitivity). Thus, we focused on the specificity score (i.e. the fraction of annotated proteins in a family that shares a common Pfam keyword versus all the proteins in the family). Table 2 lists PL70 families with a moderate size of 100–300 proteins each, all having a maximal purity (specificity score of 1.0). These families account for fundamental processes that are shared by all insects such as the various aspects of membrane trafficking and secretion. Other functions that are covered by all the analysed species include oxygen transfer and a collection of enzymatic activity. For all 3437 Pfam keywords the average specificity is 0.89 and the median is 0.93 (Figure 3).

**Table 2.** Pure cluster (Specificity of 1.0) for ProtoBug PL70 clusters having 100–300 proteins each

PFAM name	PFAM accession	Average length	No. of annotion^a^	No. of unannotation	No. of proteins	No. of clusters
CD36 family	PF01130	385.8	211	0	211	1
Eukaryotic-type carbonic anhydrase	PF00194	214.8	206	0	206	1
Astacin (Peptidase family M12A)	PF01400	174.0	177	0	177	1
Hemocyanin, all-alpha domain	PF03722	107.0	147	6	153	1
Hemocyanin, ig-like domain	PF03723	235.0	146	7	153	1
Hemocyanin, copper containing domain	PF00372	254.6	143	10	153	1
Sulfatase	PF00884	338.9	143	0	143	1
Snf7	PF03357	163.2	131	0	131	2
Innexin	PF00876	292.9	129	0	129	1
Inositol monophosphatase family	PF00459	238.9	116	0	116	1
Synaptobrevin	PF00957	73.3	107	7	114	1
Regulated-SNARE-like domain	PF13774	76.8	49	65	114	1
pfkB family carbohydrate kinase	PF00294	248.7	98	14	112	2
emp24/gp25L/p24 family/GOLD	PF01105	175.7	103	0	103	1
D-isomer specific 2-hydroxyacid dehyd., catalytic	PF00389	286.7	86	14	100	1
D-isomer specific 2-hydroxyacid dehy., NAD binding	PF02826	168.2	97	3	100	1

^a^Annotation according to Pfam keywords.

We implemented in ProtoBug Database and Webserver the notion of a correspondence score (CS) to assess the purity and coverage. The CS for a specific cluster and a given keyword is a measure for the matching between the two. Formally, let us fix a cluster C in ProtoBug and a keyword K (from a specific source such as Pfam). Let c be the set of proteins in cluster C and let k be the set of proteins in the system annotated by keyword K. We define the CS as:

$$\text{CS(C,K)=}\frac{\left|c\cap k\right|}{\left|c\cap k\right|}$$

The score for a given cluster on keyword K ranges from 0 (no correspondence) to 1 (the cluster C is comprised of all the proteins with keyword K). The CS values are used as a quality measure for the automatic and the curation-based approaches. For example, we consider the distribution of CS value over all clusters that meet a minimal size of a family. In order to obtain a biologically relevant view of the hierarchy, we applied several tests and set a minimal threshold for clusters that are enriched with coherent biological information.

### Features associated with high-quality clusters

We monitored the family’s purity in view of the associated attributes to estimate the success of the automatic annotation task. This assessment is the basis for a guideline supporting the manual and experimental curation effort. Toward this task, we look at a paired match of the ProtoBug family (4504 families, ≥10 proteins) and a Pfam keyword. The number of species in each cluster and the additional quantitative properties of the families are recorded. In this ProtoBug family centric view, some families appear multiple times. For example, ProtBug 531099 (90 proteins) associate with 5 Pfam keywords: the Phosphatidylinositol-specific phospholipase C of X and Y domains show high specificity (0.94). However, the keyword ‘Protein of unknown function’ (DUF1154) is associated with only 27 proteins. This led to a poor specificity for DUF1154 (0.3). Thus, from the family perspective, the average specificity is 0.84 where the unannotated proteins are not counted. The specificity score drops to 0.72 once the unannotated proteins are considered false positives.

**Figure 4.**
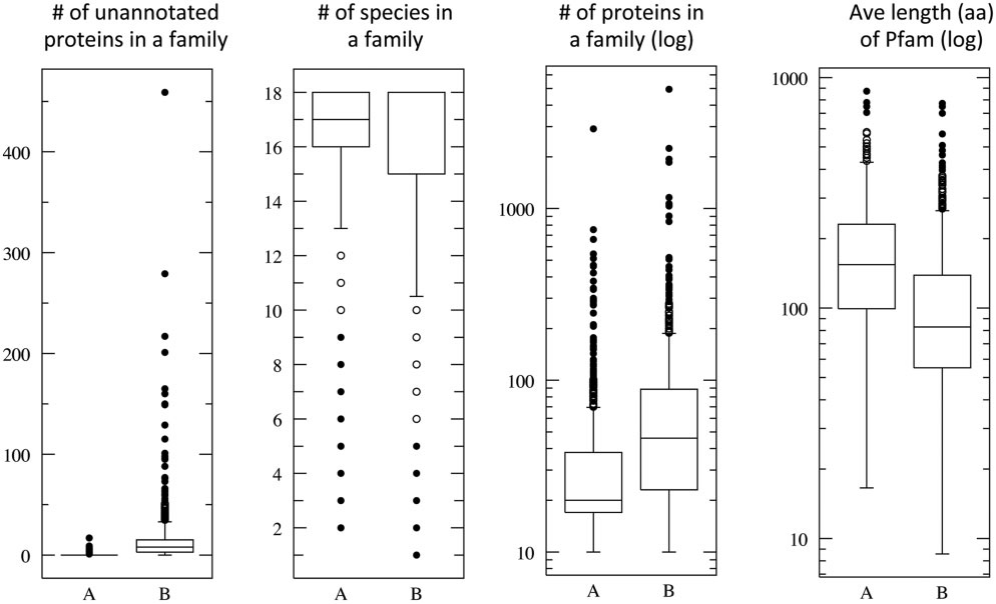
Quantitative attributes of ProtoBug families. Each panel summarizes the statistics for families according to annotation purity. All ProtoBug PL70 families (4504 with ≥10 proteins each) were ranked by the CS and the top and bottom 800 families (18%) are defined as group A and B, respectively. The statistics is presented as Plotbox with the bottom and top of the box shows the first and third quartiles, and the line inside the box shows the median. The whiskers cover the extreme 5% of the quartiles and the outliers are indicated by the dots. Note that the scale for some of the attributes is logarithmic.

Figure 4 shows the statistical distribution of the ProtoBug families that cover the highest (Figure 4, marked A) and lowest (Figure 4, marked B) values for 800 families (18% of 4504 families, ≥10 proteins). In the case of the Pfam domain/family, the average length is 178 (median 154 aa) and 110 (median 82.7 aa), for groups A and B, respectively. This significant difference supports the view that the size of the domain is one of the attributes toward a successful annotation inference. We had shown that the hierarchical algorithm is preferable for single domain proteins and for proteins carrying simple domain compositions (20). All the attributes show significant statistics with *P* < 0.001 for each of the panels (based on *t*-test, Figure 4).

The pure families (group A, Figure 4) are also characterized by being mostly represented by 18 and 17 species. ProtoBug ID 552854 is a family characteristic for ‘7tm odorant receptor’ with 1630 proteins and no false positives. Despite the size of the family, it is confirmed to be a genuine insect specific. Indeed, ProtoBug ID 552854 does not include *D. pulex* proteins. In all the cases that only 17 species were included in the family, the proteins from *D. pulex* were missing.

### Annotations’ gain in protein families

Within a family, the unannotated proteins are assumed to share the same function as the annotated proteins. We refer to such assignment as ‘annotation gain’. To avoid functional inference with a minimal support, automatic inference was restricted to families with a predetermined level of purity (>0.2 for any specific Pfam keyword). Based on this criterion, we were able to associate Pfam keywords to 57% of all unannotated proteins. This ‘guilt by association’ approach was applied for 77 988 sequences. For example, 123 unannotated proteins that belong to the ‘7tm odorant receptor’ family (ProtoBug ID 552854) can be safely annotated. Similarly, ‘cytochrome P450’, a crucial enzyme in the electron transfer that catalyses the oxidation of organic substances, together with 55 unannotated proteins forms a family of 1749 protein without any false positives (ProtoBug ID 553578). Many other families support the safe annotation inference.

Interesting instances for manual curation task are associated with proteins that were not assigned any other Pfam keyword, and families having little or no false annotations. Table 3 lists a sample of families with a minimum of 40 proteins, a false positive rate that is <5% and a domain average length that is >75 amino acids. Inspecting the table suggests safe annotations for hundreds of proteins. The gain in annotation applies for enzymes, receptors and structural functions. Interestingly, the majority of these families are not represented by all species. The same scale of annotation gain applies to families denoted by ‘Domain/Protein of Unknown Function’ (DUFs). As a guideline for experimental and manual curation task for complete proteomes, we proposed to set filters according to the parameters studied in Figure 4 (e.g. the Pfam domain average length).

**Table 3.** Annotation gain in ProtoBug PL70 clusters having <40 proteins each

Family ID	Pfam acc (PF)	Pfam name	No. of gain	No. of TP	No. of FP
552114	13 359	DDE superfamily endonuclease	142	316	6
550859	1757	Acyltransferase family	109	254	0
553707	75	RNase H	32	105	4
552319	640	Phosphotyrosine interaction domain (PTB/PID)	23	115	2
529077	2949	7tm Odorant receptor	38	85	0
552416	2145	Rap/ran-GAP	29	91	2
546351	6477	Protein of unknown function (DUF1091)	34	71	1
549834	3175	DNA polymerase type B, organellar and viral	37	63	3
549781	149	Calcineurin-like phosphoesterase	24	65	0
551188	722	Glycosyl hydrolases family 16	20	62	0
553621	11 018	Pupal cuticle protein C1	20	61	0
552272	380	Ribosomal protein S9/S16	13	36	0
541523	12 736	Cell-cycle sustaining, positive selection	13	34	0
520563	5050	Methyltransferase FkbM domain	12	35	0
552405	115	Cytochrome C and Quinol oxidase polypeptide I	10	31	0

### Seeking stable families by following a tree branch

The hierarchical property of the ProtoBug tree raises the challenge of finding a most informative level in the ProtoBug tree that will represent reliable and stable families. The PL70 was selected as a preferable level for partition the data to families.

**Figure 5.**
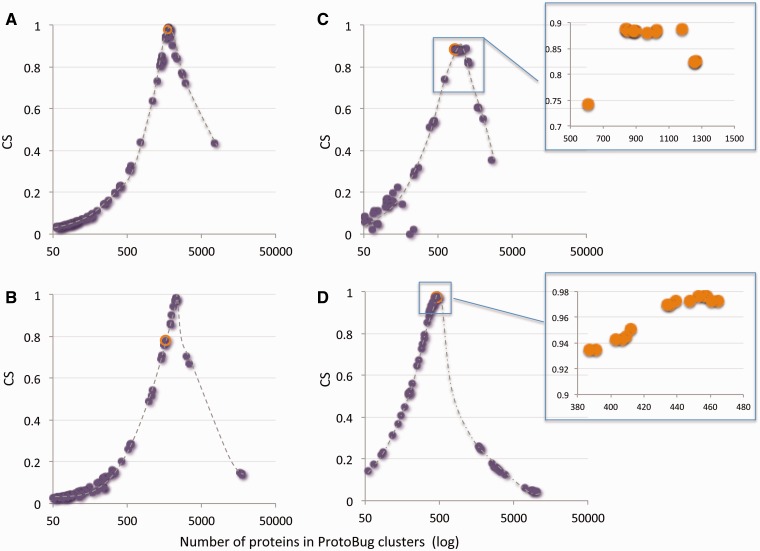
A keyword-centric view for ProtoBug families according to CS and the number of proteins. Representatives of Pfam keywords are: **(A)** Cytochrome P450; **(B)** Ligand-gated ion channel; **(C)** 7tm odorant receptor; **(D)** Cadherin domain. Each plot shows the 100 clusters with the highest CSs versus the cluster size (log scale). In most instances the PL70 family and the maximal CS for this keywords coincides (orange symbol in A–D). Insets for C and D show a zoom for the top 15 clusters. For all the keywords, a sharp drop in CS and a substantial increase in the size of the family mark the deterioration in the cluster quality towards the root of the Protobug tree.

Figure 5 shows 4 of such families for the 100 top families according to the CS score. Several observations are evident for most of the families: (i) the PL70 families capture the maximal CS for the selected keyword. (ii) From the perspective of the CS score, there are numerous clusters that have a similar performance, suggesting a set of stable clusters (Figure 5C and D, insets). (iii) All clusters show a drastic drop in their CS score that occurs in late stages of the clustering. (iv) Information on the consistency of functional family is captures within the top 100 clusters (Figure 5) and for the majority of the cases within the top 20 clusters (in view of the CS score).

For a selected keyword, following the evolvement of a family identifies ‘interim-stable clusters’ along the process. These interim-stable clusters often coincide with a partition to subfamilies. Families that contain multiple subfamilies are observed by having a not trivial fitting curve (not shown).

## Website properties

**Figure 6.**
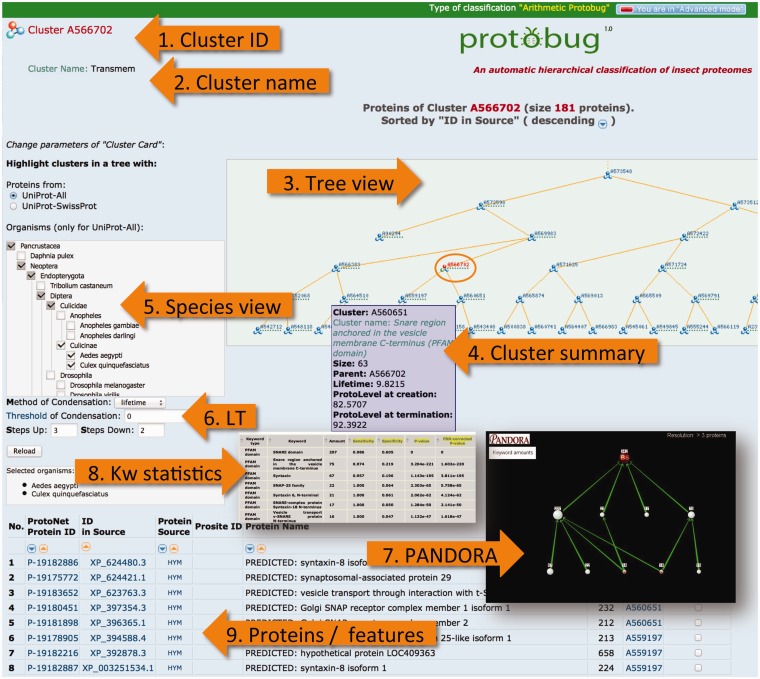
ProtoBug cluster page and several viewers from the simplified and advanced modes. The advanced mode is selected at the top right corner of the page. The cluster A566702 includes 181 proteins. A cluster is uniquely identified by its ID (1). Cluster name (2) is provided for clusters that show a minimal degree of consistency with the different resources for keyword (Pfam, Phobius, Clantox and Taxonomy). Tree viewer (3) is sensitive to the selection of the species (5) and the compression of the tree according to the LT (6). Family annotation is analysed using PANDORA viewer (7) and statistical significance (8). The proteins of the clusters are listed (9) with their immediate attributes (length, source and association to their child clusters).

Many features on the ProtoBug website are based on the generic platform on the ProtoNet tree. ProtoNet was designed for all protein sequences irrespectively of their taxa or completeness of the proteomes. We describe the capacity of ProtoBug platform. Some will be operated only in the ‘advanced’ mode (Figure 6). A Cluster page is shown at the mode of ‘viewing the proteins’ (Figure 6, proteins/features).

### Browsing cluster by keyword

Clusters’ names are available for browsing (Figure 6, Cluster name). One can choose a keyword of interest and view all the clusters that are named by it. For example, there are 4380 clusters in ProtoBug with a keyword of ‘7tm odorant receptor’. The total number of proteins with this keyword is 1930. We provide a tabular summary for all the 4380 clusters sorted by the associated CS score. Detailed listing of True positives, True negatives, False positive and False negatives for the selected keyword can be used for an off-line analysis. In ProtoBug there are ∼6300 families that are named by at least one Pfam keyword (excluding singletons).

### Summary of a cluster

Each ProtoBug cluster carries a large number of properties that include the source of protein, the position in the ProtoBug tree and the LifeTime (LT). The later defines as the difference between the termination and creation time of the cluster. The PL determines the granularity. In addition, the fraction of proteins that are named putative/hypothetical is reported. We provide a list of all these numerical properties in a summary table (Figure 6, Cluster summary).

### Tree resolution

The *ProtoBrowser* page zooms in on the tree in the vicinity of the cluster that is being analysed. A selected branch is shown in the context of related neighboring branches. The user hovers the mouse over a cluster to display essential (minimal) information such as the cluster size. An example of such *ProtoBrowser* tree view is shown (Figure 6, Tree view). ProtoBug is constructed as a binary tree. However, the user can apply the LT filter to accept proteins that meet the value of the selected LT parameter. In the ‘Advanced mode’, the user can select to investigate any level of the clustering tree (PL0–PL100).

### Species tree viewer

The user can select to view the ProtoBug tree for a subset of the 18 complete proteomes. The tree viewer highlights the selected branches (Figure 6, Species view). This option can be reset at any step of the analysis. The branches that include proteins from an organism (or a clade) become visible; though all ‘faded’ clusters can still be analysed. By activation the species viewer, the partition of the proteins in the cluster according to the selected organisms is provided. For example, the ProtoBug ID 544862 (397 proteins) includes transporters from the Major Facilitator Superfamily from all 18 analysed species. However, there are twice as many proteins from *T. castaneum* with respect to *A. mellifera* and almost 2.5 folds more than found in mosquitoes.

### Navigation in an advance mode

The advance mode provides user control on the parameters for visualization and navigation. The user can choose to activate the ProtoBrowser at a different resolution. In a simplified mode two levels above and below the selected cluster (red font in the tree) are shown by default. In the ‘advanced mode’, the number of layers to be presented is a user-selected parameter (Figure 6). By moving up the tree one observes how the cluster grows in size and becomes more diverse. The user can change the tree resolution by modifying the parameters of the tree condensation protocol. The parameter of LT is controlled. A value of zero is applicable for a binary tree (Figure 6, LT). Other capacity of the ‘advanced mode’ allows retrieving the clusters at any selected PL. A careful biological interpretation of the clusters is beyond the scope of this research.

### Multiple annotation sources

ProtoBug 1.0 includes the assignment of Pfam keywords (using the PfamScan protocol) as the major functional annotation resource. Evidently, the annotations of protein families are not limited to a single expert system. We applied Phobius predictor (21) to tag proteins for the presence of the transmembrane domains (TMDs) and signal peptide. About ∼20% proteins from the analysed proteomes are positively predicted by Phobius. Additional annotations concern a unique subset of secreted proteins that resemble ion channel blockers and cell modulators. These are positively predicted by ClanTox (CLassifier of ANimal TOXins) (22). Clantox identifies 0.7% of the ProtoBug proteins.

### Integration of annotations

A functional analysis of a family is performed using the PANDORA (23) visualization. PANDORA platform allows in-depth analysis of ProtoBug clusters by supporting the direct export from the cluster page. PANDORA allows assessing the relatedness and overlapping of any of the multiple assignment of Pfam keyword to the family members (it is applicable to any type of annotation). The protein family is forwarded to the PANDORA analysis tool. The results from the PANDORA tool include: (i) The statistical significance of the keywords that are assigned to the proteins (Figure 6, KW statistics) (23). (ii) A visualization of the inter-relations of the keywords (Figure 6, PANDORA).

### Towards function—a user’s test case

We illustrate a routine use of ProtoBug platform. The starting point is a sequence named hypothetical protein KGM_11510 from the butterfly, *Danaus plexippus* (24). Applying the function ‘Paste your sequence*’* with the default parameters finds this sequence in ProtoBug ID 481476 (17 out of the 21 proteins are named ‘synaptosomal-associated protein’). The cluster name is SNAP-25 (CS score 0.96). Navigating in the tree reveals the interconnection to a group of 16 related sequences that belong to SNAP-29, another synaptosomal-associated protein (19 proteins, ProtoBug 491190). ‘Climbing’ in the ProtoBug tree identified a cluster of 16 uncharacterized proteins (ProtoBug ID 502509) that cannot be assigned to any Pfam or other annotation. Based on the reliability of the connections (not shown) and using a structural modeling approach, we confirm this cluster as synaptosomal associated that carries the characteristics of t-SNARE domains. The higher level in the ProtoBug tree (PL90) suggests a natural partition of two stable clusters with 118 proteins (ProtoBug ID 559197) and 63 proteins (ProtoBug ID 560651). ProtoBug ID 559197 includes limited types of syntaxin proteins (Syntain 6, 8, 10) while ProtoBug ID 560651 accounts for the Golgi SNAP receptor complex (with proteins carry features of v-SNARE and t-SNARE). A stable cluster of 181 proteins contains t- and v-SNARE proteins. The merge of this cluster and additional stable one that consists mainly of syntaxins (161 proteins, ProtoBug ID 566383) creates a unified SNARE-related cluster with 342 proteins (ProtoBug ID 569983). The reliability of the superfamily as a superfamily of SNARE Arthropods is evident (CS of 0.69). Note that ∼50% of the proteins carry non-informative names (e.g. species identifier number, hypothetical). Among them, ∼50 sequences explicitly named ‘putative uncharacterized protein’.

## Maintenance and Updating

ProtoBug 1.0 incorporated improvements that will benefit automation for future updates and release versions. ProtoBug will be updated once a year. A partition in UniProt to the sections of UniProt/Swiss-Prot and UniProt/TrEMBL was implemented. This will allow the users to focus, as needed, on all the proteins or on a subset that are manually curated. Future releases will incorporate additional annotation resources. To provide the user with a control over the confidence level, the annotations evidence (e.g. experimental, inferred) will be added based on the scheme adopted by the GO consortium. The next version of ProtoBug will include additional complete genomes that are underrepresented (with emphasize on butterfly and moth).

## References

[1] Weinstock G.M. Robinson G.E. Gibbs R.A.*.* (2006) Insights into social insects from the genome of the honeybee *Apis mellifera*. Nature, 443, 931–949. 17073008 10.1038/nature05260 PMC2048586

[2] Wurm Y. Wang J. Riba-Grognuz O.*.* (2011) The genome of the fire ant *Solenopsis invicta*. Proc. Natl Acad. Sci. USA, 108, 5679–5684. 21282665 10.1073/pnas.1009690108 PMC3078418

[3] Brady S.G. Schultz T.R. Fisher B.L.*.* (2006) Evaluating alternative hypotheses for the early evolution and diversification of ants. Proc. Natl Acad. Sci. USA, 103, 18172–18177. 17079492 10.1073/pnas.0605858103 PMC1838725

[4] Loewenstein Y. Raimondo D. Redfern O.C.*.* (2009) Protein function annotation by homology-based inference. Genome Biol., 10, 207. 19226439 10.1186/gb-2009-10-2-207 PMC2688287

[5] Magrane M. Consortium U. (2011) UniProt Knowledgebase: a hub of integrated protein data. Database, 2011, bar009. 21447597 10.1093/database/bar009 PMC3070428

[6] Finn R.D. Mistry J. Tate J.*.* (2010) The Pfam protein families database. Nucleic Acids Res., 38, D211–D222. 19920124 10.1093/nar/gkp985 PMC2808889

[7] Barrell D. Dimmer E. Huntley R.P.*.* (2009) The GOA database in 2009—an integrated Gene Ontology Annotation resource. Nucleic Acids Res., 37, D396–D403. 18957448 10.1093/nar/gkn803 PMC2686469

[8] Hunter S. Apweiler R. Attwood T.K.*.* (2009) InterPro: the integrative protein signature database. Nucleic Acids Res., 37, D211–D215. 18940856 10.1093/nar/gkn785 PMC2686546

[9] Kaplan N. Linial M. (2006) ProtoBee: hierarchical classification and annotation of the honey bee proteome. Genome Res., 16, 1431–1438. 17065614 10.1101/gr.4916306 PMC1626645

[10] Rappoport N. Linial N. Linial M. (2013) ProtoNet: charting the expanding universe of protein sequences. Nat. Biotechnol., 31, 290–292. 23563419 10.1038/nbt.2553

[11] Radivojac P. Obradovic Z. Brown C.J.*.* (2003) Prediction of boundaries between intrinsically ordered and disordered protein regions. Pac. Symp. Biocomput., 216–227. 12603030

[12] Sasson O. Vaaknin A. Fleischer H.*.* (2003) ProtoNet: hierarchical classification of the protein space. Nucleic Acids Res.,31, 348–352. 12520020 10.1093/nar/gkg096 PMC165543

[13] Loewenstein Y. Portugaly E. Fromer M.*.* (2008) Efficient algorithms for accurate hierarchical clustering of huge datasets: tackling the entire protein space. Bioinformatics, 24,i41–i49. 18586742 10.1093/bioinformatics/btn174 PMC2718652

[14] UniProt Consortium. (2011) The Universal Protein Resource (UniProt) in 2010. Nucleic Acids Res., 38, D142–D148. 10.1093/nar/gkp846 PMC2808944 19843607

[15] Munoz-Torres M.C. Reese J.T. Childers C.P.*.* (2011) Hymenoptera Genome Database: integrated community resources for insect species of the order Hymenoptera. Nucleic Acids Res., 39, D658–D662. 21071397 10.1093/nar/gkq1145 PMC3013718

[16] Colbourne J.K. Pfrender M.E. Gilbert D.*.* (2011) The ecoresponsive genome of *Daphnia pulex*. Science, 331,555–561. 21292972 10.1126/science.1197761 PMC3529199

[17] Altschul S.F. Madden T.L. Schaffer A.A.*.* (1997) Gapped BLAST and PSI-BLAST: a new generation of protein database search programs. Nucleic Acids Res, 25, 3389–3402. 9254694 10.1093/nar/25.17.3389 PMC146917

[18] Sasson O. Kaplan N. Linial M. (2006) Functional annotation prediction: all for one and one for all. Protein Sci., 15, 1557–1562. 16672244 10.1110/ps.062185706 PMC2242553

[19] Fischer S. Brunk B.P. Chen F.*.* (2011) Using OrthoMCL to assign proteins to OrthoMCL-DB groups or to cluster proteomes into new ortholog groups. Curr. Protoc. Bioinformatics, Chapter 6, Unit 6.12 1 11–19. 21901743 10.1002/0471250953.bi0612s35 PMC3196566

[20] Rappoport N. Karsenty S. Stern A.*.* (2012) ProtoNet 6.0: organizing 10 million protein sequences in a compact hierarchical family tree. Nucleic Acids Res., 40, D313–D320. 22121228 10.1093/nar/gkr1027 PMC3245180

[21] Kall L. Krogh A. Sonnhammer E.L. (2007) Advantages of combined transmembrane topology and signal peptide prediction-the Phobius web server. Nucleic Acids Res., 35, W429–W432. 17483518 10.1093/nar/gkm256 PMC1933244

[22] Naamati G. Askenazi M. Linial M. (2009) ClanTox: a classifier of short animal toxins. Nucleic Acids Res., 37, W363–W368. 19429697 10.1093/nar/gkp299 PMC2703885

[23] Rappoport N. Fromer M. Schweiger R.*.* (2010) PANDORA: analysis of protein and peptide sets through the hierarchical integration of annotations. Nucleic Acids Res., 38, W84–W89. 20444873 10.1093/nar/gkq320 PMC2896089

[24] Zhan S. Merlin C. Boore J.L.*.* (2011) The monarch butterfly genome yields insights into long-distance migration. Cell, 147, 1171–1185. 22118469 10.1016/j.cell.2011.09.052 PMC3225893

